# Animal Models of Idiopathic Inflammatory Myopathies: Bridging Mechanistic Insights and Clinical Translation

**DOI:** 10.3390/ijms27156697

**Published:** 2026-07-27

**Authors:** Guanyuan Wang, Shaoxin Cui, Lin Yang, Karl J. A. McCullagh, Cuiqing Ma, Aijing Liu

**Affiliations:** 1 Department of Rheumatology and Immunology, The Second Hospital of Hebei Medical University, Shijiazhuang 050000, China; guanyuanwanghmu@163.com (G.W.); cuishx491568126@126.com (S.C.); lyn2684@163.com (L.Y.); 2 Hebei Medical University-National University of Ireland Galway Stem Cell Research Center, Hebei Medical University, Shijiazhuang 050017, China; 3 Hebei International Joint Research Center on Rheumatic Diseases, Shijiazhuang 050000, China; 4 Discipline of Physiology, School of Pharmacy & Medical Sciences, Human Biology Building, University of Galway, H91 W5P7 Galway, Ireland; karl.mccullagh@universityofgalway.ie; 5 Key Laboratory of Immune Mechanism and Intervention on Serious Disease in Hebei Province, Department of Immunology, Hebei Medical University, Shijiazhuang 050017, China; 6 Hebei Research Center for Stem Cell Medical Translational Engineering, Shijiazhuang 050017, China

**Keywords:** idiopathic inflammatory myopathies, animal models, pathogenesis, immunopathology, translational medicine

## Abstract

Idiopathic inflammatory myopathies (IIMs) are a heterogeneous group of autoimmune diseases with wide variation in pathogenesis, clinical presentation, and serological profiles, leading to substantial differences in diagnosis, classification, treatment response, and prognosis among patients. Although decades of progress have been made in understanding myositis-specific autoantibodies and molecular pathology, important gaps remain in elucidating disease mechanisms and identifying effective therapeutic targets. Therefore, animal models that recapitulate key characteristics of human IIMs provide an indispensable platform for addressing current limitations in mechanistic research and promoting translational studies. This review comprehensively discusses animal models related to the major clinical–serological subtypes of IIMs, including polymyositis-like T-cell-mediated models, dermatomyositis models, inclusion body myositis models, antisynthetase syndrome models, and immune-mediated necrotizing myopathy models. Particular attention is given to model construction, pathological phenotypes, dominant immune mechanisms, therapeutic applications, and translational limitations. By organizing current models according to subtype-related pathological and immunological features, this narrative review aims to provide a clearer framework for selecting appropriate experimental systems and to facilitate more precise, mechanism-driven myositis research.

## 1. Introduction

Idiopathic inflammatory myopathies (IIMs) are a heterogeneous group of systemic autoimmune rheumatic diseases, characterized primarily by sterile inflammation of skeletal muscle and immune-mediated myofiber damage. The major clinical subtypes include dermatomyositis (DM), immune-mediated necrotizing myopathy (IMNM), inclusion body myositis (IBM), and antisynthetase syndrome (ASyS) [1,2,3]. These subtypes exhibit distinct epidemiological profiles, including differential age at onset, sex distribution, clinical manifestations, and long-term prognosis [4]. Although classification criteria and serological testing have improved, considerable overlap in clinical and pathological features remains, suggesting that IIMs are better regarded as a spectrum of related disorders rather than a single disease entity [2,4].

Skeletal muscle serves as the primary target tissue in IIMs. This tissue specificity may be related to immune responses targeting muscle-associated antigens and the inherent susceptibility of the local immune microenvironment, which together contribute to persistent muscle injury [5]. Even though skeletal muscle is the core site of pathological damage, severe cases of IIMs often present multiorgan involvement, affecting the skin, lungs, gastrointestinal tract, heart, and joints [1]. Interstitial lung disease (ILD) and cutaneous manifestations represent the most prevalent extramuscular phenotypes, which are predominantly detected in patients with DM and ASyS [1].

The identification of myositis-specific autoantibodies (MSAs) and the application of integrated clinical, serological, and pathological classification have greatly improved our understanding of the clinical and mechanistic heterogeneity of IIMs [2,6], and have also prompted a critical reappraisal of the traditional diagnosis of polymyositis (PM) as a diagnostic category. With the refinement of serological and pathological classification, the diagnosis of PM has become substantially more restrictive, and many cases previously classified as PM are now reassigned to IMNM, ASyS, overlap myositis, or sporadic IBM [7,8]. Accordingly, in this review, we use “PM-like” as an operational, mechanism-oriented descriptor for models of cell-mediated myofiber injury—including those historically designated as PM models and others better viewed as experimental autoimmune myositis (EAM) models—rather than as a claim that they faithfully reproduce PM as a currently recognized standalone entity. Notably, the pathological features of cytotoxic CD8^+^ T-cell invasion into major histocompatibility complex class I (MHC-I)-expressing non-necrotic myofibers are not unique to historical PM but are also central inflammatory features of sporadic IBM [9]. Therefore, models such as EAM, C-protein-induced myositis (CIM), and the human peripheral blood mononuclear cell (hPBMC)-based humanized model are most useful for studying selected forms of T-cell-mediated myofiber injury, rather than for modeling PM or any other current clinical subtype in its entirety [10,11,12]. In general, IIMs involve dysregulation of innate and adaptive immunity and eventually lead to immune-mediated myofiber damage [5].

Different IIM subtypes display well-characterized immunopathological signatures. DM is mainly driven by over-activation of the type I interferon (IFN-I) pathway, as evidenced by myxovirus resistance protein A (MxA) overexpression, MHC-I upregulation, capillary damage, and membrane attack complex (MAC) deposition within muscle tissue [1]. In IMNM, anti-signal recognition particle (anti-SRP) and anti-3-hydroxy-3-methylglutaryl coenzyme A reductase (anti-HMGCR) autoantibodies are strongly associated with complement-mediated myofiber necrosis [13]. ASyS is usually associated with anti-aminoacyl-tRNA synthetase antibodies (e.g., anti-Jo-1, anti-PL-7, anti-PL-12, anti-EJ, and others) and exhibits perimysial pathology, MHC-I upregulation, complement deposition, and frequent multisystem involvement, including pulmonary and articular manifestations [14].

**Figure 1:**
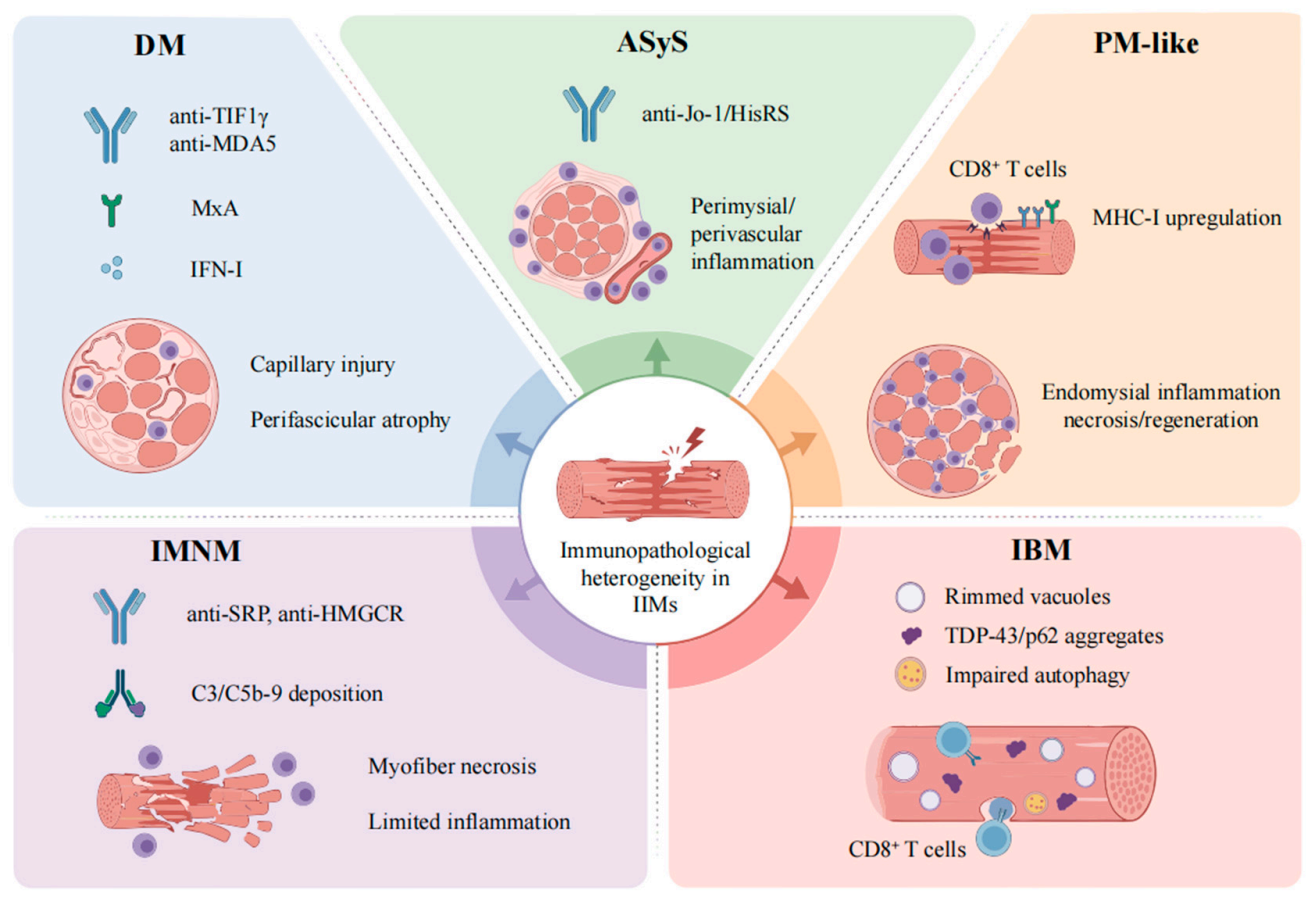
Schematic representation of **i**mmunopathological heterogeneity across major idiopathic inflammatory myopathy (IIM) subtypes. This schematic summarizes the key autoantibodies, immune-related pathways, and histopathological features of major human IIM subtypes, including dermatomyositis (DM), antisynthetase syndrome (ASyS), PM-like T-cell-mediated myositis, inclusion body myositis (IBM), and immune-mediated necrotizing myopathy (IMNM). It is important to note that the schematic does not imply that any corresponding animal model recapitulates the full spectrum of each subtype. DM is characterized by type I interferon (IFN-I) pathway activation, myxovirus resistance protein A (MxA) expression, capillary injury, and perifascicular atrophy. ASyS is associated with anti-aminoacyl-tRNA synthetase (anti-ARS) autoantibodies, particularly anti-Jo-1, and perimysial/perivascular inflammation. PM-like myositis highlights T-cell-mediated muscle injury accompanied by major histocompatibility complex class I (MHC-I) upregulation. IBM combines cytotoxic T-cell infiltration with degenerative features, including rimmed vacuoles and intracellular protein aggregates. IMNM is defined by antibodies against signal recognition particle (SRP) or 3-hydroxy-3-methylglutaryl-coenzyme A reductase (HMGCR), complement deposition, myofiber necrosis, and relatively limited inflammation. These subtype-specific immunopathological differences provide a pathological and immunological basis for selecting and interpreting corresponding animal models.

Sporadic inclusion body myositis (sIBM) is characterized by infiltration of highly differentiated cytotoxic cluster of differentiation 8-positive (CD8^+^) T cells, production of autoantibodies against cytosolic 5′-nucleotidase 1A (cN1A; also known as NT5C1A), and degenerative changes including TAR DNA-binding protein 43 (TDP-43) and p62/sequestosome 1 (SQSTM1) protein aggregation, alongside endoplasmic reticulum stress [9]. In addition, dysfunction of regulatory T cells and abnormalities of immune checkpoint pathways such as T-cell immunoreceptor with Ig and ITIM domains (TIGIT) and programmed cell death protein 1 (PD-1) may further weaken immune tolerance, maintain inflammatory responses, and promote chronic disease progression [15]. As illustrated in Figure 1, these subtype-specific immunopathological features translate into distinct histopathological correlates, highlighting the complexity of the molecular immune mechanisms in IIMs and providing a basis for the development of more precise, subtype-guided therapeutic treatment strategies.

The treatment of IIMs remains a substantial clinical challenge. Although glucocorticoids and conventional immunosuppressants can alleviate symptoms and retard disease progression in some patients, their long-term use is often associated with adverse effects, including osteoporosis, infections, and metabolic abnormalities. Moreover, a considerable proportion of patients continue to experience disease recurrence, inadequate treatment responses, or persistent functional disability [16,17]. Therefore, there is a continuing and unmet need to develop novel therapeutic strategies with immunomodulatory and tissue-repairing capacities.

In this context, animal models provide an important experimental platform for investigating disease mechanisms and facilitating clinical translation. Models related to different IIM subtypes can, to varying degrees, reproduce key pathological features, including immune dysregulation, myofiber damage, metabolic aberrations, and multiorgan involvement. This narrative review offers a subtype- and mechanism-oriented overview of currently available IIM animal models, classifying them by their links with major clinical–serological subtypes and pathogenic mechanisms, rather than solely by induction methodology. Within this framework, self-antigen-driven models, such as transcription intermediary factor 1γ (TIF1γ), melanoma differentiation-associated gene 5 (MDA5), histidyl-tRNA synthetase (HisRS/Jo-1), SRP, and HMGCR models, can be interpreted in relation to their respective DM, ASyS, and IMNM disease subtypes. Table 1 provides a comprehensive overview of the major IIM subtypes, their representative clinicopathologic features, and the corresponding animal models discussed in this review. The subsequent sections discuss these models in greater detail, focusing on their construction, pathological phenotypes, research applications, and translational relevance.

## 2. PM-like T-Cell-Mediated Myositis Models

### 2.1. EAM Model

The EAM model remains one of the most widely used animal models for studying PM-like, T-cell-mediated inflammatory myopathies, because it can recapitulate, to a certain extent, immune-mediated myofiber injury [10]. EAM is usually induced by immunizing animals with muscle-associated autoantigens emulsified with adjuvants [18]. Early protocols often used repeated immunization with muscle homogenate and complete Freund’s adjuvant (CFA) [19], while current methods more commonly involve subcutaneous injection of partially purified myosin emulsified with CFA over 3–4 weeks, often in combination with pertussis toxin (PT) to enhance immune activation [10,20,21]. In recent years, optimization of immunization regimens, including simplification of booster immunization procedures, has further improved the reproducibility and efficiency of disease induction [22]. It should be noted that some strains, such as SJL/J mice, have congenital dysferlin defects that may affect disease progression, and this genetic background should therefore be considered when interpreting experimental outcomes [23].

Histopathologically, EAM is mainly characterized by marked skeletal muscle inflammation, manifesting as myofiber necrosis and regeneration, accompanied by an inflammatory infiltrate dominated by CD4^+^ T cells and monocytes/macrophages, with relatively sparse B cell involvement [10]. The inflammatory lesions are typically distributed in the intrafascicular, endomysial, and interfascicular regions, and collectively they more closely mimic the predominant pathological features of cell-mediated immune injury observed in human PM or partial overlap myositis cases; however, they lack the typical perifascicular atrophy and complement-mediated microangiopathy of DM [24]. In addition, affected animals frequently exhibit splenomegaly, suggesting systemic immune activation [25]. At the molecular level, activation of IFN-I signaling promotes upregulation of MHC-I expression on myofibers, thereby favoring CD8^+^ T-cell recruitment and cytotoxic injury [26,27]. Moreover, IFN-β-mediated oxidative stress and mitochondrial dysfunction may also further aggravate muscle injury [24].

Recent studies have further expanded the mechanistic scope of the EAM model, indicating that it is not only involved in the adaptive immune response but also closely related to innate immunity and tissue damage signals. Aberrant T-cell costimulation and its downstream signaling can promote pro-inflammatory T helper cell differentiation [15], whereas the corresponding negative regulatory pathways contribute to the maintenance of immune homeostasis [28]. Concurrently, oxidative stress and mitochondrial dysfunction may interact with myeloid cell activation to jointly amplify tissue damage [29].

Beyond skeletal muscle inflammation, EAM-related or modified myositis-ILD models have also been employed to explore pulmonary involvement in IIMs. Polarization of pro-inflammatory macrophages and the excessive formation of neutrophil extracellular traps (NETs) may further aggravate inflammation through innate immune signaling and may be associated with extramuscular manifestations such as ILD [30,31,32,33,34]. Mechanistically, NET-related signaling activates the Toll-like receptor 9 (TLR9)-interferon regulatory factor 3 (IRF3) axis in alveolar epithelial cells, and TLR9 agonists in combination with muscle homogenate further promote NET formation. These findings suggest that the NETs-TLR9 feed-forward loop may constitute an innate immune bridge between muscle autoimmunity and IIM-related ILD [35].

Consistent with this proposed mechanism, pharmacological inhibition of NET formation with the peptidylarginine deiminase (PAD) inhibitor Cl-amidine attenuated EAM-associated interstitial lung lesions and pulmonary NET accumulation [31]. Conversely, co-administration of the TLR9 agonist ODN2395 with muscle homogenate induced a NET-rich myositis-associated ILD phenotype [35]. However, to our knowledge, direct genetic testing of this pathway in EAM or related myositis-associated ILD models—for example, using *Tlr9*^−/−^ or peptidyl arginine deiminase 4 (*Padi4*)^−/−^ mice—has not yet been reported. Nevertheless, EAM is amenable to genetic manipulation, as illustrated by the protection of global interleukin-6 (*Il6*)^−/−^ mice from myosin-induced myositis and by muscle-specific nuclear factor erythroid 2-related factor 2 (*Nrf2*) deletion, which reduced CD8^+^ T-cell infiltration and attenuated muscle weakness [29,36]. Based on these mechanistic insights, EAM and its modified variants have been extensively utilized to evaluate a variety of targeted therapeutic and non-pharmacological interventions. Representative interventions, molecular targets, and preclinical results are shown in Table 2.

In summary, the EAM model is a valuable tool for studying T-cell-mediated myositis and its underlying pathogenic mechanisms, as well as for preclinical treatment evaluation. However, because it does not fully reproduce the subtype heterogeneity or the chronic disease course of human IIMs, its translational relevance still has some limitations.

### 2.2. C-Protein-Induced Myositis (CIM) Model

The CIM model was first established by Kohyama et al. as a modified variant of the classic EAM model. This model is induced by immunization with skeletal muscle C protein, an explicit autoantigen, in combination with CFA and PT [11]. In early experiments using Lewis rats, repeated intramuscular immunization successfully induced muscle inflammation and overt muscle weakness. Subsequently, a mouse model of CIM based on a recombinant human C protein fragment was further established. However, these approaches remain constrained by a lack of reproducibility and high cost [45]. Histopathologically, CIM recapitulates key features of CD8^+^ T-cell-mediated immune myopathy, including dense inflammatory infiltrates within myofiber bundles, myofiber necrosis and regeneration of myofibers, and significant upregulation of MHC-I on myofibers, suggesting that myofibers may actively participate in local antigen presentation and immune amplification [11,46,47].

Serum creatine kinase (CK) can serve as a complementary biochemical marker of muscle injury in CIM. In a therapeutic study using recombinant C-protein fragment-induced CIM, vehicle-treated mice developed progressive loss of grip strength, increased serum CK activity, and inflammatory muscle pathology characterized by mononuclear cell infiltration and higher histopathological scores. Treatment with the selective immunoproteasome inhibitors ONX 0914 or KZR-616 preserved grip strength, reduced serum CK activity, and alleviated inflammatory muscle lesions [48]. However, a direct association between CK levels and antibody responses has not been established; moreover, earlier CIM studies observed unexpectedly high CK levels even in some healthy mice, potentially owing to uncontrollable hemolysis [45]; therefore, CK measurements should be interpreted in conjunction with functional, histopathological, and antigen-specific immune endpoints.

From a mechanistic perspective, the CIM model provides important clues for understanding CD8^+^ T-cell-mediated muscle injury and its regulatory mechanisms. The functional heterogeneity of the PD-1^+^ CD8^+^ T-cell population, together with inducible programmed death-ligand 1 (PD-L1) expression on myofibers, suggests the existence of an endogenous immunomodulatory circuit within muscle tissue [49]. Meanwhile, costimulatory signaling pathways and regulated cell death mechanisms are also implicated in disease progression. Modulation of cluster of differentiation 80/86 (CD80/CD86)-mediated costimulatory signaling or inhibition of necroptotic cell death has been reported to significantly reduce muscle inflammation and tissue damage [47,50]. These mechanistic studies provide a basis for evaluating targeted therapeutic strategies. Representative interventions, including immunoproteasome inhibition, mitochondrial transfer, and metabolic cytoprotection strategies, are further summarized in Table 3 [48,51,52].

Overall, the CIM model provides a useful platform for investigating antigen-specific T-cell-mediated myositis and related therapeutic approaches, although its stability and capacity to replicate disease heterogeneity remain limited.

### 2.3. hPBMC-Based Humanized Mouse Model

To overcome the limitations of traditional mouse models in recapitulating human disease, researchers have developed a humanized myositis model via adoptive transfer of hPBMCs into immunodeficient NOD *scid* gamma (NSG) mice. NSG mice carry the *scid* mutation in conjunction with a null mutation of the *Il2rg* gene encoding the common γ-chain of the IL-2 receptor; consequently, they lack mature T and B cells, and, given that γ-chain-dependent IL-15 signaling is essential for natural killer (NK)-cell development, they also lack functional NK cells. Although residual host myeloid cells are present, macrophage phagocytic activity, dendritic-cell maturation and function, and complement activity are impaired on the NOD genetic background [53]. This profound host immunodeficiency permits efficient engraftment of human cells; however, engrafted human T cells can also attack murine tissues and drive xenogeneic graft-versus-host disease (GVHD). In the original study, mice were euthanized upon manifestation of GVHD-like signs or on day 20 post-transfer, with no fixed onset time for GVHD being reported [12].

Intravenous administration of hPBMCs induced a reproducible myositis phenotype, including elevated CK and aspartate aminotransferase (AST) levels, upregulated expression of muscle injury-related genes such as vascular cell adhesion molecule 1 (*Vcam1*), intercellular adhesion molecule 1 (*Icam1*), and serum amyloid A1 (*Saa1*), and pronounced infiltration of human CD8^+^ T cells in skeletal muscle. These histopathological and molecular changes closely resembled those observed in patients with PM. Mechanistically, disease pathogenesis in this model was largely dependent on CD8^+^ T cells, as their depletion prior to transplantation significantly attenuated both systemic and local muscle lesions. The infiltrating CD8^+^ T cells showed an activated cytotoxic phenotype, with expression of PD-1, human leukocyte antigen-DR (HLA-DR), and granzyme B, indicative of effector/memory T-cell characteristics. Furthermore, transcriptome analysis showed significant enrichment of gene signatures associated with PM, and the majority of upregulated genes overlapped with disease-related pathways, suggesting that this model partially recapitulates key immunopathological features of PM at both cellular and molecular levels [12].

Beyond its mechanistic value, this humanized model may provide a translationally relevant platform for studying human myositis. Treatment with clinically established immunosuppressants, such as prednisolone and tacrolimus, significantly reduced muscle inflammation and serum muscle enzyme levels, supporting its applicability for preclinical drug evaluation. Compared with the classical EAM and CIM models, the hPBMC-based system introduces human immune effector cells, which can reduce the confounding effects of interspecies differences to a certain extent and more closely mimic CD8^+^ T-cell-mediated muscle injury. In addition, this model can exhibit extramuscular manifestations such as pulmonary involvement, enhancing its relevance for modeling the systemic nature of IIMs [12].

## 3. DM Models

### 3.1. TIF1γ-Induced Myositis (TIM) Model

The TIM model was established in female C57BL/6 mice aged 8–10 weeks. Recombinant human TIF1γ protein emulsified with CFA was administered intradermally into the back skin and footpads of mice once weekly for 4 weeks, with PT co-administered in combination at the last immunization to enhance the immune response. This regimen can induce a relatively stable myositis phenotype of TIF1γ-specific myositis within 2 weeks, with a modeling success rate of approximately 70–83%; some animals may show persistent pathological changes [54]. Phenotypically, the model predominantly affects proximal muscles, including the hamstrings and quadriceps femoris, and mimics some of the key histopathological features of anti-TIF1γ-positive dermatomyositis (DM), such as myofiber atrophy and necrosis, focal perifascicular atrophy, and endomysial mononuclear inflammatory cell infiltration [55]. In addition, significant upregulation of sarcolemmal MHC-I expression and marked induction of interferon-stimulated genes such as myxovirus resistance 1 (*Mx1*) are also consistent with the typical pathological features of clinical DM.

Mechanistically, disease pathogenesis in the TIM model is primarily mediated by CD8^+^ cytotoxic T cells. Interference with MHC-I antigen presentation, impairment of cytotoxic effector function, or antibody-mediated depletion of CD8^+^ T cells significantly attenuated disease severity. Conversely, adoptive transfer of CD8^+^ T cells into untreated recipient mice was sufficient to induce myositis. An IFN-I signature was also observed in this model, as shown by reduced disease severity in type I interferon receptor (IFNAR)-deficient mice, as well as increased expression of IFN-related genes in affected muscle tissue. This model is one of the few experimental systems capable of simulating anti-TIF1γ-related DM-like pathological changes and can be used for related mechanistic studies and therapeutic evaluation. However, this model does not fully reproduce the complete immunopathological profile of human DM, and the expression pattern of IFN-related genes differs from that observed in patients. The lesions are dominated by CD8^+^ T-cell infiltration, which does not fully reflect the more complex immune cell composition observed in clinical DM; therefore, its translational relevance remains limited [54].

### 3.2. MDA5-Induced ILD Model

The MDA5-ILD model is established using a “two-hit” strategy, combining autoimmunity against MDA5 with viral mimicry-mediated lung injury. In this model, mice are first immunized with recombinant murine MDA5 protein emulsified in CFA to induce antigen-specific adaptive immune responses, followed by intranasal administration of polyinosinic-polycytidylic acid [poly(I:C)] to mimic viral double-stranded RNA exposure and promote pulmonary inflammation [56]. This sequential treatment strategy simulates the proposed pathogenic concept of anti-MDA5-positive DM, wherein environmental viral stimulation may further promote disease development on the background of pre-existing autoimmunity [57]. At the phenotypic level, this model develops severe acute lung injury, which gradually progresses to persistent peribronchiolar inflammation and fibrotic remodeling, thereby recapitulating key features of rapidly progressive ILD (RP-ILD), including sustained inflammation, impaired injury resolution, and collagen deposition [56,58].

Mechanistically, disease progression in this model is primarily driven by CD4^+^ T cells rather than humoral immunity. T-cell deficiency prevents disease development, whereas B cell deficiency does not confer protection. Selective depletion of CD4^+^ T cells, but not CD8^+^ T cells, significantly attenuated pulmonary fibrosis, suggesting that CD4^+^ T cells are the principal pathogenic effector cells. At the molecular level, sustained activation of IFN-I signaling and prolonged expression of interleukin-6 (IL-6) appear to jointly contribute to inflammatory amplification and fibrotic progression; accordingly, blockade of IL-6 receptor signaling significantly alleviates lung pathology, suggesting that IL-6 may represent a potential therapeutic target in this model.

Compared with other IIM models, this model is distinctive in its integration of autoantigen-specific immune responses with lung-dominant fibrotic outcomes. However, this model is fundamentally lung-centric in design, with pulmonary fibrosis as the primary readout and no modeling of skeletal muscle involvement. It therefore does not recapitulate the systemic, multiorgan phenotype of anti-MDA5-positive disease in humans, which may include cutaneous manifestations, arthritis, and variable muscle involvement alongside ILD. It also remains an experimental simulation of viral triggering and exhibits certain discrepancies in cytokine profiles compared with those observed in patients [56].

## 4. IBM Models

Experimental models of IBM have been developed to clarify its complex pathogenesis. According to their dominant pathological features, current models can be broadly divided into two mechanistic categories: those centered on proteotoxic stress and protein aggregation, and those designed to reflect autoimmune-mediated muscle damage. The former emphasizes degenerative pathology associated with intracellular protein accumulation, whereas the latter mainly captures immune-mediated myofiber injury. These two groups are complementary rather than interchangeable in modeling the dual pathological nature of IBM.

### 4.1. Protein Aggregation-Based IBM Models

Transgenic mice overexpressing β-amyloid precursor protein (βAPP) were among the first models used to reproduce protein aggregation-related features of IBM [59,60]. In these models, the muscle creatine kinase (MCK) promoter drives skeletal-muscle-specific expression of human βAPP, resulting in several IBM-like pathological changes, including amyloid-β (Aβ)40 and Aβ42 deposition, intrafiber vacuole formation, and centrally located myonuclei, features that indicate ongoing myofiber injury and regeneration [61]. To improve disease relevance, the myosin light chain (MLC)-βAPP mouse model was subsequently developed, in which the MLC1/3 promoter restricts βAPP expression to adult fast-twitch fibers, which more closely reflects the adult-onset pattern of sIBM. The model exhibits Aβ aggregation, abnormal tau accumulation, muscle weakness, and disrupted calcium homeostasis, supporting its utility for studying impaired excitation–contraction coupling in sIBM [62].

Several additional models have extended this protein aggregation-based framework. A muscle-specific TDP-43 transgenic model relies on a tetracycline-inducible system to express human TDP-43 lacking its nuclear localization signal, leading to cytoplasmic accumulation of phosphorylated and insoluble inclusions, accompanied by progressive muscle dysfunction. Notably, the prion-like propagation behavior of TDP-43 aggregates points to a potential self-amplifying process of protein aggregation in IBM [63]. The mutant gelsolin model (D187N), which was originally associated with Finnish-type familial amyloidosis, also contributes to this category by inducing amyloid deposition, endoplasmic reticulum (ER) stress, and progressive muscle degeneration, thereby linking abnormal proteolysis with myopathic pathology [64,65]. Another relevant model is based on RNF5 overexpression and draws attention to dysfunction of ER-associated degradation (ERAD). In this model, inducible accumulation of RNF5 triggers ER stress and activates unfolded protein response pathways, eventually producing IBM-like pathological features, including rimmed vacuoles, Congo red-positive inclusions, and progressive myofiber damage [66].

In addition to genetic manipulation, pharmacological disruption of proteostasis has been employed as an alternative approach to model IBM-related degenerative pathology. The chloroquine (CQ)-induced model uses chronic lysosomal dysfunction to inhibit autophagic flux. Following intraperitoneal CQ administration, rats develop sustained lysosomal alkalinization, which reduces hydrolase activity and interferes with autophagosome-lysosome fusion. As a result, autophagy-related markers, including beclin-1, BNIP3, and LC3-II, accumulate abnormally, whereas lysosomal proteins such as LAMP-2 and cathepsin L are reduced [67,68]. Histologically, slow-twitch muscles, such as the soleus, show rimmed vacuoles, β-amyloid deposition, and myofiber atrophy, thereby reproducing part of the degenerative spectrum of sIBM. This model is therefore useful for analyzing how lysosomal-autophagic failure contributes to aggregate formation and for evaluating autophagy-modulating therapeutic strategies.

### 4.2. Immune-Mediated IBM Models

Immune-mediated models are primarily employed to reproduce the inflammatory and autoimmune components of IBM. The cN1A peptide immunization model is induced by repeated administration of murine cN1A peptides in combination with CFA and PT, eliciting antigen-specific immune responses in C57BL/6 mice [69]. This model induces anti-cN1A autoantibodies and recapitulates several characteristic immunopathological changes, including CD8^+^ T-cell infiltration into the endomysium, invasion of non-necrotic myofibers, and upregulation of MHC-I expression. At the molecular level, the accumulation of p62 and LC3 indicates impaired autophagy and proteolytic dysfunction, suggesting a close association between immune activation and disrupted protein degradation pathways. Functionally, affected animals exhibit reduced motor performance, further supporting the model’s relevance to IBM-related disease progression [69].

Collectively, current IBM models recapitulate selected components rather than the integrated clinical–pathological continuum of human sIBM. Protein aggregation-based transgenic systems, including βAPP, TDP-43, gelsolin, and RNF5 models, together with the chloroquine-induced lysosomal dysfunction model, primarily model individual proteostatic or degenerative pathways, such as protein aggregation, ER stress, and impaired autophagy [59,60,61,62,63,64,65,66,67,68]. Although informative for these specific mechanisms, they do not fully reproduce the age-associated and slowly progressive coexistence of cytotoxic inflammation, myofiber degeneration, and mitochondrial abnormalities characteristic of human sIBM [70,71]. The cN1A immunization model partly complements these approaches by incorporating autoantibody responses, CD8^+^ T-cell infiltration, and proteostatic abnormalities, but does not reproduce the sustained degenerative progression of established sIBM [69]. The human muscle xenograft model retains both inflammatory and degenerative features and is currently the closest approximation of human IBM pathology; however, it precludes functional and behavioral assessment, and some pathological changes may overlap with graft-versus-host-like responses [70,71,72]. Thus, no currently available model fully integrates the proteotoxic, immune, mitochondrial, and long-term functional dimensions of sIBM.

## 5. ASyS Models

ASyS is characterized by the concomitant occurrence of myositis and ILD in the presence of autoantibodies against synthetases, most commonly HisRS/Jo-1. Katsumata et al. established an antigen-specific ASyS-like model by immunizing C57BL/6 congenic (B6.G7) and NOD congenic (NOD.Idd3/5) mice with recombinant murine Jo-1, or its amino-terminal 151-amino acid fragment (MA/MBP), emulsified in CFA. A notable feature of this model is its species-specific immune response: immunization with murine Jo-1 elicited robust B and T-cell responses preferentially targeting self-murine Jo-1, whereas the corresponding human protein generated antibodies largely restricted to human Jo-1. This finding suggests that conformational determinants may be important in breaking immune tolerance [73].

Histopathologically, MA/MBP-immunized mice developed focal muscle inflammation with perimysial, perivascular and endomysial infiltrates in approximately 70% of animals by 8 weeks, along with highly reproducible interstitial lung inflammation characterized by perivascular, peribronchiolar, and alveolar lymphocytic infiltrates. In NOD.Idd3/5 mice, full expression of the muscle–lung phenotype required additional costimulation via tumor necrosis factor receptor superfamily member 4 (OX40). Mechanistically, class-switched immunoglobulin G (IgG) responses, progressive epitope spreading, and antigen-specific CD4^+^ T-cell proliferation collectively support a T-cell-dependent response against native Jo-1. This phenotype recapitulates the clinical observation that ILD occurs in more than half of Jo-1 antibody-positive patients, who often present with relatively mild myositis. Principal limitations of this model include its dependence on self-protein immunization to bypass tolerance, the focal nature of myositis, and the requirement for OX40 costimulation in certain genetic backgrounds [73].

In the standard HisRS model, innate immune activation is strictly MyD88-dependent and is mediated in part by redundant TLR2/TLR4 signaling [74]. Pharmacological TLR7/8 activation with R848 induces persistent local inflammation and inflammatory spread to uninjected muscle; type I IFN is required for the latter and for the prolonged autoantibody response [26]. However, *Tlr7* and the endosomal solute carrier family 15 member 4 (SLC15A4)-TLR adaptor interacting with SLC15A4 on the lysosome-IRF5 module implicated in lupus-associated IFN-I signaling have not been genetically tested in this model [75]; their targeted deletion would be required to determine whether this pathway contributes causally to IFN-I activation and disease amplification.

## 6. IMNM Models

To elucidate the pathogenic role of anti-SRP and anti-HMGCR autoantibodies in IMNM, Bergua et al. established animal models that recapitulate the main pathological features of IMNM through passive transfer and active immunization strategies. The investigators passively transferred patient plasma or purified IgG into wild-type (WT) C57BL/6 mice, *Rag2*^−/−^ mice deficient in T and B cells, and *C3*^−/−^ mice deficient in complement. They found that both WT and *Rag2*^−/−^ mice developed marked muscle weakness and necrotizing myositis, whereas pathological changes were significantly attenuated in *C3*^−/−^ mice. Of note, supplementation with human complement in *C3*^−/−^ mice restored the originally attenuated disease phenotype, thereby confirming the key role of the classical complement pathway in IMNM pathogenesis. In addition, active immunization with recombinant SRP54 or HMGCR protein in combination with adjuvants induced sustained autoantibody production and IMNM-like pathological changes [76], providing further support for the classification of IMNM as an antigen-specific, antibody-mediated disease.

Histopathological examination of affected skeletal muscle revealed that this model reproduced a necrotizing myositis phenotype, with widespread myofiber necrosis and regeneration but only limited inflammatory cell infiltration. The infiltrating cells consisted predominantly of macrophages, with only scattered CD4^+^ T cells, which is consistent with the pathological hallmark of IMNM, namely “necrosis with minimal inflammation.” Deposition of C5b-9, C3, and human IgG was observed on the sarcolemma and necrotic myofibers, and IgG and C3 clearly colocalized with the target antigens SRP54 and HMGCR on the muscle membrane. Further in vitro evidence indicated that patient-derived anti-SRP and anti-HMGCR IgG could bind directly to their corresponding sarcolemmal antigens and induce complement-dependent cytolysis as well as macrophage-mediated phagocytosis. Given the high conservation and abundant expression of SRP54 and HMGCR in both human and mouse skeletal muscle, these models appear capable of reproducing the central pathological features of human IMNM with relatively good stability [76].

This human IgG-transfer IMNM model also has clear value for translational studies, particularly for evaluating therapies directed at pathogenic antibodies or complement activation. In this model, early administration of the C5 inhibitor zilucoplan significantly reduced complement deposition and preserved muscle strength. However, its protective effect was most evident when treatment was initiated before disease onset, whereas the therapeutic benefit was more limited after disease establishment. These findings suggest that complement-targeted therapy may have a critical therapeutic window and may need to be initiated early in the disease course, for example, when serum muscle enzyme levels are markedly elevated but before obvious muscle weakness appears [77].

In contrast, the neonatal Fc receptor (FcRn) antagonist Efgartigimod was effective in both prophylactic and therapeutic settings and promoted muscle regeneration and functional recovery. By reducing pathogenic autoantibody levels in both the circulation and muscle tissue, efgartigimod provides a rationale for clinical evaluation of FcRn blockade in antibody-associated IIMs, including IMNM. A related clinical trial (NCT05523167) has been initiated to evaluate this therapeutic need [78]. Nevertheless, several limitations should be noted. The natural disease course of this model has not been fully characterized, which restricts its use for studying the chronic progression of human IMNM. Moreover, disease induction depends on exogenous complement reconstitution and therefore may not fully reflect endogenous complement activation as it occurs in patients [76,77,78].

## 7. Cross-Model Limitations and Translational Interpretation of IIM Animal Models

### 7.1. Shared Limitations Across Current IIM Animal Models

Many inducible models reproduce an acute or subacute, largely monophasic disease course over a few weeks, rather than the chronic and, in certain subtypes, relapsing course characteristic of human IIMs [10,11,54]. Consequently, clinical phenomena such as relapse after initial response, treatment-refractory weakness, and cumulative myofiber loss underlying fixed functional disability are poorly captured. Chronic structural remodeling, including persistent muscle fibrosis and fatty replacement, is also incompletely represented in most currently available models [4]. This mismatch is particularly evident in IBM, an age-associated, slowly progressive disorder with both degenerative and inflammatory features that is generally refractory to immunosuppressive therapy and whose characteristic pathology accumulates over years [9]. Current IBM models capture isolated components of this process: aggregation-based and chloroquine models recapitulate selected degenerative pathways, whereas cN1A immunization primarily captures autoimmune features without sustained degenerative progression [59,60,61,62,63,64,65,66,67,68,69].

A further shared limitation is the incomplete representation of systemic and subtype-specific organ involvement in human IIMs. Most models focus predominantly on skeletal muscle. Even when extramuscular manifestations are included, they are often captured only partially rather than as the full multisystem phenotype. The MDA5-ILD model is lung-centric, with persistent pulmonary inflammation and fibrosis as its principal readouts, while no detectable muscle inflammation or cutaneous lesions are present [56]. The HisRS/Jo-1 model provides a muscle–lung phenotype, but the myositis is focal and requires immunization with self-protein [73]. The humanized hPBMC model can also show pulmonary involvement [12]. Moreover, models generated in a single inbred strain and through immunization with one defined autoantigen represent highly standardized experimental settings. Although they can reproduce selected subtype-related mechanisms, they cannot fully capture the clinical heterogeneity of IIMs, in which MSA profile, disease stage, genetic background, age, and previous treatment may all influence phenotype and therapeutic response [4,6].

Many commonly used IIM models primarily reproduce T-cell-mediated tissue injury. EAM, CIM, the humanized hPBMC model, TIM, and the MDA5-ILD model principally reflect CD4^+^- or CD8^+^-driven damage [10,11,12,54,56]. As a result, sustained subtype-specific B-cell and plasma-cell responses, endogenous pathogenic autoantibody production, complement-dependent injury, and the full diversity of innate immune populations are less consistently represented across the current model repertoire. This limits preclinical evaluation of therapies whose primary targets lie outside T-cell pathways. Several systems partially address these gaps: modified EAM-ILD models incorporate NET-associated inflammation and macrophage-related pathways [30,31,32,33,34,35]; HisRS/Jo-1 immunization elicits autoreactive B- and T-cell responses, IgG class switching, epitope spreading, and muscle–lung inflammation [73], while the related HisRS model highlights reciprocal myeloid–T-cell interactions linking innate and adaptive immunity, with MyD88 signaling in both T cells and myeloid cells contributing to full disease expression [79]. The cN1A model combines anti-cN1A autoantibody responses with CD8^+^ T-cell infiltration and proteostatic abnormalities [69], and anti-SRP/anti-HMGCR IMNM models reproduce autoantibody- and complement-dependent injury, with patient-derived IgG inducing necrotizing weakness in *Rag2*^−/−^ mice and disease attenuated in C3-deficient mice [76]. Nevertheless, these systems incorporate selected humoral or innate features rather than sustained and integrated B-cell and plasma-cell pathology. A model specifically designed to interrogate intrinsic B-cell function together with persistent plasmablast and plasma-cell responses is still lacking, despite the relevance of these mechanisms to subtype-specific autoantibody production and emerging B-cell- and plasma-cell-directed therapies in IIMs [80].

These limitations are further shaped by how the models are constructed. In active-immunization models, adjuvants are used to break immune tolerance and may generate phenotypes that reflect experimentally amplified immune activation rather than spontaneous disease initiation [10,11,54,56,73]. Host genetic background can also influence model interpretation: the dysferlin defect in SJL/J mice may confound muscle pathology, while the HisRS-associated muscle–lung phenotype is background-dependent, with OX40 costimulation required for full phenotypic expression in certain congenic strains [23,73]. Species differences in immune and complement biology further constrain the interpretation of antibody- and complement-mediated injury models [76,77,78]. hPBMC-based humanized models partly mitigate these differences by introducing human immune effector cells but introduce potentially confounding graft-versus-host responses [12,81].

Biological sex is a potentially important but underexamined source of variability in IIM animal models. Sex distribution differs across IIM subtypes, and sex chromosomes, sex hormones, and sex-biased immune pathways can influence autoimmune susceptibility and inflammatory responses [82]. Although sex-dependent differences in disease severity and immune phenotypes have been reported in other induced autoimmune models, including experimental autoimmune myocarditis and experimental autoimmune encephalomyelitis [83,84], the influence of sex on disease induction, immune responses, and treatment effects has rarely been systematically examined in current IIM models. Future studies should report animal sex, age, and strain, justify single-sex designs, and provide sex-stratified analyses where adequately powered [85].

### 7.2. Translational Interpretation and Model Selection

The heterogeneity of IIMs renders the pursuit of a single representative model unrealistic, and no existing system reproduces the full clinical, serological, and pathological spectrum of human IIMs [2,4,6]. A myositis phenotype or histological resemblance to human muscle does not in itself establish translational relevance; what matters is whether a model’s dominant mechanism is aligned with the research question, therapeutic target, and experimental endpoint. Evaluated on this basis rather than on overall similarity, the models reviewed here occupy distinct and largely complementary niches (Table 4).

Clinical resemblance and mechanistic value should be considered separately across models. Some map more directly onto a defined clinical–serological subset: TIM reproduces selected pathological features of anti-TIF1γ-associated DM [54,55], HisRS/Jo-1 immunization models reproduce antigen-specific muscle–lung autoimmunity in ASyS [73], and passive-transfer or active-immunization IMNM models reproduce autoantibody- and complement-mediated necrotizing injury [76]. Others are used principally to dissect defined pathogenic processes: EAM and CIM for T-cell-mediated inflammatory muscle injury [10,11,49], and protein-aggregation IBM models for discrete proteostatic pathways, including aggregate accumulation, impaired autophagy, and endoplasmic reticulum dysfunction [59,60,61,62,63,64,65,66,67,68]. These systems remain important for identifying pathogenic pathways and testing pathway-directed interventions, but are best interpreted at the level of the mechanism they reproduce rather than as complete substitutes for the corresponding human diseases.

The same principle guides selection by therapeutic target. An intervention is best tested in a model whose dominant mechanism, summarized for each system in Table 4, matches the pathway it engages and whose primary endpoint can assess the intended effect. EAM and CIM are most informative for agents directed at T-cell activation, costimulation, inflammatory amplification, or myofiber death [10,29,33,47,48,51,52]. Therapies acting through human CD8^+^ T-cell effectors can be assessed in the hPBMC-NSG model [12,81], whereas antibody-lowering, complement-inhibitory, and FcRn-directed approaches are most appropriately examined in IMNM transfer models [76,77,78]. Questions concerning anti-MDA5-associated ILD, IFN/IL-6 signaling, or pulmonary fibrosis are best addressed in the MDA5-ILD model, with treatment effects judged primarily by pulmonary rather than muscle endpoints [56,58]. Strategies targeting protein homeostasis, autophagy, or ER stress are better suited to degenerative IBM models [59,63,66,67,68].

The IMNM transfer model illustrates the importance of matching disease stage as well as mechanism to the therapeutic question. C5 inhibition was effective when administered before disease onset but showed limited functional benefit after established injury [77]. This stage-dependent preclinical pattern was consistent with the lack of significant efficacy observed with zilucoplan in a phase 2 trial of established IMNM [86], underscoring that disease stage, as well as dominant mechanism, should align with the intended therapeutic setting.

Finally, a positive result in one model should not be extrapolated to IIMs as a whole or to subtypes it does not represent [4]. Suppression of inflammation in EAM or CIM does not establish efficacy against the autoantibody- and complement-driven injury of IMNM [10,11,13,76]. More broadly, benefit observed in an organ-restricted model should not be assumed to extend to unmodeled organ manifestations. The strongest preclinical inferences arise when the therapeutic target, the model’s dominant mechanism, the relevant disease stage and organ endpoint, and the intended patient subtype are concordant, with key findings ideally corroborated in a complementary model. Used as mechanistically defined, deliberately selected tools rather than interchangeable surrogates, current models can provide a more reliable foundation for translational research in IIMs.

## 8. Conclusions and Future Perspectives

Animal models have become indispensable tools for dissecting the complex immunopathology of IIMs and for bridging mechanistic research with clinical translation. Existing models, ranging from early generalized systems to more recent subtype-oriented approaches, have substantially advanced our understanding of T-cell-mediated immunity, complement activation, and targeted therapeutic strategies [10,35]. However, most current models still do not fully reproduce the chronic progression, interindividual variability, subtype-specific complications, or extramuscular manifestations of human IIMs. Their predominantly acute or subacute disease course, coupled with interspecies differences, continues to constrain their direct translational applicability.

Future efforts should prioritize the development of models that more closely reflect clinical disease, including chronic and heterogeneous systems, genetically humanized mice, and multiorgan systems capable of capturing clinically relevant comorbidities. The integration of induced pluripotent stem cell-derived muscle models [87], microphysiological systems, and advanced imaging technologies is expected to further improve the predictive value of preclinical platforms. In the longer term, continued refinement of animal models, combined with a clearer “subtype–mechanism–intervention” framework, should strengthen bidirectional translation between basic research and clinical practice and support the development of more individualized therapeutic strategies for IIMs.

## Figures and Tables

**Table 1:** Overview of major IIM subtypes and representative animal models.

IIM Subtype	Key Autoantibody or Antigen	Main Pathogenic Features	Representative Clinicopathologic Features	Representative Animal Models	Representative Strains and Genetic Evidence
PM-like models	Usually seronegative; myosin or C protein	T-cell-mediated myofiber injury	Endomysial inflammation; myofiber necrosis and regeneration; MHC-I upregulation	EAM; CIM; hPBMC-based humanized model	SJL/J (EAM); Lewis rat/C57BL/6 (CIM); NSG (hPBMC model). *Il6*^−/−^ and muscle-specific *Nrf2* deletion attenuate EAM.
DM models	TIF1γ; MDA5	IFN-I-associated immune injury	Perifascicular atrophy; MxA/MHC-I upregulation; cutaneous or lung involvement	TIM; MDA5-ILD model	C57BL/6. IFNAR deficiency attenuates TIM.
IBM models	cN1A/NT5C1A; protein aggregation-related targets	Cytotoxic inflammation and proteotoxic stress	Asymmetric weakness; rimmed vacuoles; protein aggregates; endomysial inflammation	βAPP, TDP-43, gelsolin, RNF5, chloroquine-induced, and cN1A immunization models	Various transgenic mouse backgrounds; rats (chloroquine model); C57BL/6 (cN1A immunization).
ASyS models	HisRS/Jo-1	Antigen-specific muscle–lung autoimmunity	Myositis with ILD; perimysial/perivascular inflammation; antisynthetase antibody response	HisRS/Jo-1 immunization models	B6.G7/NOD.Idd3/5 (Jo-1); C57BL/6-derived mutant lines (HisRS). MyD88 dependence and combined *Tlr2/Tlr4* deletion have been genetically demonstrated.
IMNM models	SRP; HMGCR	Autoantibody- and complement-mediated necrosis	Severe weakness; myofiber necrosis with minimal inflammation; C5b-9 deposition	Passive IgG/plasma transfer; active SRP54 or HMGCR immunization	C57BL/6, *Rag2*^−/−^, and *C3*^−/−^ mice. C3 deficiency attenuates passive-transfer disease.

ASyS, antisynthetase syndrome; βAPP, β-amyloid precursor protein; C3, complement component 3; C5b-9, terminal complement complex; CIM, C-protein-induced myositis; cN1A/NT5C1A, cytosolic 5′-nucleotidase 1A; DM, dermatomyositis; EAM, experimental autoimmune myositis; HisRS, histidyl-tRNA synthetase; HMGCR, 3-hydroxy-3-methylglutaryl-coenzyme A reductase; hPBMC, human peripheral blood mononuclear cell; IBM, inclusion body myositis; IFN-I, type I interferon; IFNAR, type I interferon receptor; IgG, immunoglobulin G; *Il6*, interleukin-6; ILD, interstitial lung disease; IMNM, immune-mediated necrotizing myopathy; MDA5, melanoma differentiation-associated gene 5; MHC-I, major histocompatibility complex class I; MxA, myxovirus resistance protein A; MyD88, myeloid differentiation primary response 88; *Nrf2*, nuclear factor erythroid 2-related factor 2; NSG, NOD *scid* gamma; PM, polymyositis; *Rag2*, recombination activating gene 2; RNF5, ring finger protein 5; SRP, signal recognition particle; SRP54, 54-kDa subunit of the signal recognition particle; TDP-43, TAR DNA-binding protein 43; TIF1γ, transcription intermediary factor 1γ; TIM, TIF1γ-induced myositis; *Tlr2/Tlr4*, Toll-like receptor 2/Toll-like receptor 4.

**Table 2:** Summary of therapeutic and non-pharmacological interventions evaluated in EAM models.

Intervention	Target/Pathway	Main Findings	Translational Value	Clinical Evidence	Ref.
Tacrolimus (FK506)	Calcineurin-dependent T-cell activation	Reduced histological muscle inflammation and ICAM-1^+^ cell infiltration in myosin-induced EAM	Supports preclinical evaluation of calcineurin-targeted immunosuppression in PM-like myositis	Evaluated in a prospective randomized open-label comparison in PM/DM-associated ILD	[37,38]
Rapamycin	mTOR; Treg/effector T-cell balance	Improved muscle strength and reduced muscle inflammation	Potential immunomodulatory and steroid-sparing strategy for PM-like myositis	Evaluated in a phase 2b randomized, double-blind, placebo-controlled trial in sIBM; the primary knee-extension strength endpoint was not met	[39]
IVIG	Autoantibodies and complement	Reduced anti-myosin antibody levels, complement deposition, and muscle lesions	Supports humoral immunity and complement as therapeutic targets	Supported by a phase 3 randomized, placebo-controlled trial in adult DM	[20,40]
NAC/ROS scavenging	Mitochondrial ROS; IFN-I-related injury	Attenuated ROS accumulation, mitochondrial dysfunction, IFN-stimulated transcripts, inflammation, and muscle weakness	Suggests mitochondrial protection as an adjunctive strategy	No published IIM-specific clinical efficacy study was identified	[24]
Colchicine	NETs and inflammasome-related injury	Reduced NET formation and alleviated lung lesions in EAM-ILD	Supports NET-targeted therapy in myositis-associated ILD	Preclinical evidence only	[33]
Muscle *Nrf2* inhibition	Muscle Nrf2-CCL5 axis	Muscle-specific *Nrf2* deficiency reduced CCL5 expression, CD8^+^ T-cell infiltration, and muscle weakness	Supports muscle Nrf2 inhibition as a potential chemokine-targeted strategy	Preclinical evidence only	[29]
Eccentric resistance training	Small HSPs; ER stress	Improved myofibrillar force and reduced ER stress without exacerbating muscle damage	Supports controlled resistance training for IIM-related muscle weakness	Randomized controlled evidence supports high-intensity resistance training in stable myositis	[41,42]
HIIT	PGC-1α; mitochondrial oxidative capacity; ER stress	Improved fatigue resistance, restored mitochondrial function, and reduced ER stress responses	Supports exercise-based rehabilitation for IIM-related muscle dysfunction	Supported by a multicenter randomized controlled trial in patients with recent-onset IIM	[43,44]

CCL5, C-C motif chemokine ligand 5; DM, dermatomyositis; EAM, experimental autoimmune myositis; EAM-ILD, experimental autoimmune myositis-associated interstitial lung disease; ER, endoplasmic reticulum; HIIT, high-intensity interval training; HSPs, heat-shock proteins; ICAM-1, intercellular adhesion molecule 1; IFN-I, type I interferon; IIM, idiopathic inflammatory myopathy; ILD, interstitial lung disease; IVIG, intravenous immunoglobulin; mTOR, mammalian target of rapamycin; NAC, N-acetylcysteine; NETs, neutrophil extracellular traps; *Nrf2*, nuclear factor erythroid 2-related factor 2; PGC-1α, peroxisome proliferator-activated receptor γ coactivator 1α; PM, polymyositis; ROS, reactive oxygen species; sIBM, sporadic inclusion body myositis; Treg, regulatory T cell.

**Table 3:** Representative therapeutic strategies evaluated in the CIM model.

Target Pathway	Intervention	Mechanism and Outcome	Corresponding Clinical Evidence/Status	Ref.
Immunoproteasome	KZR-616 (ONX 0914 analog)	Selectively inhibits the LMP7 subunit to suppress pro-inflammatory signaling, attenuating myositis severity and muscle inflammation	A completed phase 2 randomized, double-blind, placebo-controlled, crossover multicenter trial in active PM/DM (NCT04033926; *n* = 25).	[48]
Mitochondrial function	PN-101 (mitochondrial transplantation)	Restores mitochondrial bioenergetics and modulates inflammatory responses, improving muscle function and alleviating tissue inflammation	A completed prospective, open-label, dose-escalation phase 1/2a study in refractory PM/DM (NCT04976140; *n* = 9).	[51]
Necroptosis	PF1801 (GLP-1 receptor agonist)	Inhibits necroptotic signaling via the AMPK-PGAM5-Nrf2 axis, reducing myofiber death and preserving muscle integrity	A phase 2 randomized, double-blind, placebo-controlled trial in IIM has been registered (NCT05833711); no published efficacy results were identified.	[52]

PM, polymyositis; DM, dermatomyositis; GLP-1, glucagon-like peptide-1; LMP7, low molecular mass polypeptide 7; AMPK, AMP-activated protein kinase; PGAM5, phosphoglycerate mutase family member 5; *Nrf2*, nuclear factor erythroid 2-related factor 2; NCT, ClinicalTrials.gov identifier; n, number of participants.

**Table 4:** Comparative assessment of translational relevance and model selection across IIM animal models.

Model	Reproduced Human Features	Main limitations	Dominant Mechanism	Implication for Therapeutic Studies
EAM (PM-like)	Endomysial inflammation; myofiber injury; MHC-I upregulation; ILD in modified variants	Lacks a disease-defining autoantibody-complement axis and subtype heterogeneity	CD4^+^ T-cell and macrophage activation; IFN-I and innate amplification	T-cell, innate immune, and adjunctive-targeted interventions
CIM (PM-like)	CD8^+^ T-cell infiltration; necrosis/regeneration; MHC-I upregulation; antigen-specific responses	Limited chronicity and subtype specificity; variable CK readout	Antigen-specific CD8^+^ T-cell activation; PD-1/PD-L1; necroptosis	CD8^+^ T-cell-, costimulatory-, immunoproteasome-, and necroptosis-targeted therapies
hPBMC-NSG humanized model (PM-like)	Human CD8^+^ infiltration; PM-like transcriptional changes; occasional pulmonary involvement	Lacks autoantibody/complement-mediated injury and subtype-specific pathology	Human CD8^+^ effector/memory T-cell-mediated injury	Testing therapies acting on human immune effectors
TIM/TIF1γ model (DM)	Perifascicular atrophy; MHC-I upregulation; IFN-I signature	Incomplete DM immune phenotype, microangiopathy, and cutaneous disease	CD8^+^ cytotoxic T-cell- and IFN-I-dependent injury	CD8^+^ T-cell- and IFN-I-directed therapies
MDA5-ILD model (DM)	Lung inflammation, impaired resolution, and fibrosis	Does not model muscle, skin, or joint involvement	CD4^+^ T cells; IFN-I and IL-6; “two-hit” autoimmunity-viral mimicry	ILD-focused IFN/IL-6 and antifibrotic strategies
Protein-aggregation IBM models	Rimmed vacuoles; protein aggregates; autophagic defects; weakness	Lack cytotoxic inflammation and integrated sIBM pathology	Protein aggregation, ER stress, and impaired autophagy	Proteostasis-, autophagy-, and ER stress-targeted therapies
Immune-mediated IBM models (cN1A immunization; human xenograft)	Anti-cN1A autoimmunity and CD8^+^ T cell invasion; xenografts retain inflammatory and degenerative pathology	Limited sustained disease progression; xenografts lack systemic and functional readouts	Autoimmunity, CD8^+^ inflammation, and proteostatic abnormalities	Immune-targeted studies and pathological mechanism analyses
HisRS/Jo-1 immunization model (ASyS)	Muscle–lung inflammation; anti-Jo-1 responses; class-switched IgG	Focal myositis and incomplete systemic phenotype	Antigen-specific, T-cell-dependent muscle–lung autoimmunity	Studies of the muscle–lung axis and innate–adaptive immune interactions
IMNM passive-transfer/active-immunization models	Necrosis with minimal inflammation; IgG/C5b-9 deposition	Limited chronicity and sustained B-/plasma-cell pathology	Pathogenic anti-SRP/HMGCR IgG and complement-mediated injury	Antibody-lowering, complement-inhibitory, and FcRn-directed therapies

ASyS, antisynthetase syndrome; C5b-9, terminal complement complex; CIM, C-protein-induced myositis; CK, creatine kinase; cN1A, cytosolic 5′-nucleotidase 1A; DM, dermatomyositis; EAM, experimental autoimmune myositis; ER, endoplasmic reticulum; FcRn, neonatal Fc receptor; hPBMC, human peripheral blood mononuclear cell; HMGCR, 3-hydroxy-3-methylglutaryl-coenzyme A reductase; IBM, inclusion body myositis; IFN-I, type I interferon; IgG, immunoglobulin G; IL-6, interleukin-6; ILD, interstitial lung disease; IMNM, immune-mediated necrotizing myopathy; MDA5, melanoma differentiation-associated gene 5; MHC-I, major histocompatibility complex class I; NSG, NOD *scid* gamma; PD-1/PD-L1, programmed cell death protein 1/programmed death-ligand 1; PM, polymyositis; sIBM, sporadic inclusion body myositis; SRP, signal recognition particle; TIM, TIF1γ-induced myositis; TIF1γ, transcription intermediary factor 1γ.

## Data Availability

No new data were created or analyzed in this study. Data sharing is not applicable to this article.
